# Short-Training Damage Detection Method for Axially Loaded Beams Subject to Seasonal Thermal Variations

**DOI:** 10.3390/s23031154

**Published:** 2023-01-19

**Authors:** Marta Berardengo, Francescantonio Lucà, Marcello Vanali, Gianvito Annesi

**Affiliations:** 1 Department of Mechanical, Energy, Management and Transportation Engineering, Università degli Studi di Genova, Via all’Opera Pia, 15A, 16145 Genoa, Italy; 2 Department of Mechanical Engineering, Politecnico di Milano, Via La Masa, 34, 20156 Milan, Italy; 3 Department of Engineering and Architecture, Università degli Studi di Parma, Parco Area delle Scienze, 181/A, 43124 Parma, Italy

**Keywords:** structural health monitoring, unsupervised learning, environmental variations, principal component analysis, short baseline, tie-rods, beam-like structures, mahalanobis squared distance

## Abstract

Vibration-based damage features are widely adopted in the field of structural health monitoring (SHM), and particularly in the monitoring of axially loaded beams, due to their high sensitivity to damage-related changes in structural properties. However, changes in environmental and operating conditions often cause damage feature variations which can mask any possible change due to damage, thus strongly affecting the effectiveness of the monitoring strategy. Most of the approaches proposed to tackle this problem rely on the availability of a wide training dataset, accounting for the most part of the damage feature variability due to environmental and operating conditions. These approaches are reliable when a complete training set is available, and this represents a significant limitation in applications where only a short training set can be used. This often occurs when SHM systems aim at monitoring the health state of an already existing and possibly already damaged structure (e.g., tie-rods in historical buildings), or for systems which can undergo rapid deterioration. To overcome this limit, this work proposes a new damage index not affected by environmental conditions and able to properly detect system damages, even in case of short training set. The proposed index is based on the principal component analysis (PCA) of vibration-based damage features. PCA is shown to allow for a simple filtering procedure of the operating and environmental effects on the damage feature, thus avoiding any dependence on the extent of the training set. The proposed index effectiveness is shown through both simulated and experimental case studies related to an axially loaded beam-like structure, and it is compared with a Mahalanobis square distance-based index, as a reference. The obtained results highlight the capability of the proposed index in filtering out the temperature effects on a multivariate damage feature composed of eigenfrequencies, in case of both short and long training set. Moreover, the proposed PCA-based strategy is shown to outperform the benchmark one, both in terms of temperature dependency and damage sensitivity.

## 1. Introduction

Structures are naturally subject to deterioration and material degradation, which can lead to critical damage conditions. When the structural integrity is compromised, system current or future performances are affected. Thus, being able to detect damage at an early stage plays a key role in order to carry out prompt maintenance actions, preventing structural failure. This aspect has a relevant impact, first and foremost in terms of safety for the users, but also from an economic point of view. Indeed, carrying out effective maintenance actions, acting only when required, allows a better use of the maintenance resources.

The research area aiming at defining automatic damage detection strategies goes by the name of structural health monitoring (SHM) [1]. Due to the availability of advanced sensing techniques, data acquisition, computing, and information management, these strategies are mainly data-driven, i.e., they exploit data acquired by sensors on the monitored structure. Since no device directly measures damage, a crucial point is the extraction of damage sensitive quantities, or damage features, from the signals acquired by the sensors [2].

Vibration-based approaches are among the most commonly adopted approaches, as reported by many exhaustive review papers in the literature (e.g., [3,4,5,6,7]). According to these approaches, damage sensitive features are extracted from the dynamic response of the monitored structure by adopting, e.g., time series models [8,9,10] or modal analysis [11], relying on a simple assumption: damage manifests itself as a change in structural properties (e.g., a change of mass, stiffness, constraint characteristics or structural connectivity) that reflects in changes of modal parameters (i.e., eigenfrequencies, mode shapes and damping coefficients) [12]. Vibration-based approaches are also called global approaches [12], since the information that can be extracted from the response of a structure is related to the overall structural condition. This aspect comes with two significant advantages. Firstly, as opposite to local techniques, vibration-based techniques can be successfully adopted to detect damage without knowing the expected damage location in advance. Secondly, vibration-based techniques often use a limited number of sensors and the instrumentation required can be easily integrated in the monitored structure [5,13]. Vibration-based techniques, together with their practical advantages, are crucial for all those structures whose dynamic behaviour is significantly affected by damage, such as tie-rods, which are the main focus of this study.

Tie-rods are axially-loaded metallic beams used to balance lateral forces in arches and vaults of civil structures. Due to their characteristics, these slender elements undergo significant vibration levels under operational conditions, which make the adoption of vibration-based SHM techniques particularly suitable. Considering real operating tie-rods, they show a high uncertainty, generally associated to geometrical and material properties, loading conditions and constraint characteristics. Moreover, many different damage scenarios are possible and, in most cases, damage-related data are not available at the beginning of the monitoring phase. These factors make the use of supervised methods difficult and unreliable. Thus, an unsupervised learning approach becomes interesting, since damage is assessed when a statistically significant variation of the adopted vibration-based damage features is observed, with respect to a reference condition [2].

However, the main obstacle to the adoption of unsupervised learning approaches to real structures is related to the effects of environmental and operational variations [14]. Indeed, changes of environmental variables, e.g., temperature, cause changes to structural properties that can significantly increase the variability associated to vibration-based damage features [15,16]. For the specific case of tie-rods, it has been observed that this high variability can mask the effects of damage at an early stage, hampering a prompt damage detection [17,18].

In the literature of SHM, different approaches have been proposed to face the problems related to environmental and operational variations. A family of approaches is that of input-output models, which require measurements of both the environmental variables (the input) and the structural response (the output) to filter out the environmental effects through the adoption of, e.g., linear correlation models [19,20,21,22], neural networks [23,24,25] or support vector machines [26,27]. However, often not all the relevant environmental and operational variables are measured or known. For this reason, output-only approaches can be adopted to compensate environmental and operational changes, without relying on any additional measurement related to these changes.

When output-only techniques are considered, a possible approach to filter out the temperature effects is to actually include the normal variability of environmental factors in the training data and to use multivariate data with enough redundancy to remove the unwanted effects, using the data correlation structure [28]. Some recent examples of such approach can be found in the literature, based on Kalman filtering [29], Bayesian virtual sensing [30,31] and principal component analysis (PCA) [32,33,34]. One of the most popular tool is the multivariate metrics known as Mahalanobis squared distance (MSD) [35]. The MSD is used to assess when a new observation of a multivariate damage feature is an outlier with respect to a reference data set, called the baseline set. The MSD can naturally filter out the environmental variability, provided that a proper baseline set, containing the full range of environmental conditions, is adopted and a high-enough number of variables is used, to ensure some separability between the damage effects and the environmental effects [35]. Therefore, a critical aspect for MSD-based damage detection, and in general for any method relying on an exhaustive baseline, is the amount of time needed to build such a complete baseline set, representative of most of the natural variability. There are several cases, indeed, where this aspect prevents a reliable use of monitoring systems, and where methods not sensitive to changes of operational and environmental conditions are necessary to properly detect structural damage. This paper aims at solving this problem by proposing an SHM method able to filter out any change of the considered damage feature due to environmental effects, and able to work even when short training set, which is inevitably lacking in information, must be used.

Many different cases fall in this category and would benefit of an SHM method with these peculiarities; some examples are listed below:

- when a new structure is considered, the reference data acquired at beginning of the monitoring campaign refers to the healthy condition of the structure. In this case, damage detection cannot be effectively carried out until all the temperature conditions are observed, due to long-term seasonal effects. This can imply excessively long time before being able to start the actual monitoring of the structure, also resulting in the impossibility of detecting early damages;
- another critical scenario could be that of a case where an already operating structure shows a suspicious structural behaviour that suggests the installation of an SHM system, such as in the case of tie-rods of historical buildings. In this case, since damage can potentially be already ongoing, the goal would be detecting the possible evolution of the deterioration process. In such a situation, the need for a long training set represents a clear limit;
- even when a long and exhaustive training set is possible, there could be cases where the structure finds itself working in rare operating and environmental conditions, not accounted for in the training set (e.g., extreme meteorological events, different climate conditions). In these situations, an SHM method unable to filter out the effects of these changes on the damage feature would detect a structural damage/alteration, leading to a false positive.

In these scenarios, the SHM approach here proposed has a great impact with implications in many fields such as safety, maintenance and system reliability.

It is worth mentioning that another possible approach, which can be used as an alternative to the one proposed here, is that of adopting damage features which are not sensitive to environmental and operational variations [36,37]. This approach is attractive, since it directly tackles the cause of the problem. However, it is also challenging and difficult to apply, since it is hard to find vibration-based damage features showing a high sensitivity to damage and, at the same time, a low sensitivity to environmental effects. This is especially true for the structures considered here, i.e., tie-rods. Indeed, during their normal operational conditions, temperature variations cause changes in the mechanical and geometrical properties of both the tie-rod and the structure, which reflects into changes of the axial load and, thus, of the dynamic response properties. However, at the same time, other tension variations are due to deformation and displacement of the connecting walls, that may be caused by terrain crawl, subsidence of foundations or seismic events [17,38].

Tie-rods are, thus, challenging structures for SHM procedure. Most of the works in the literature related to SHM of tie-rods regard the axial-load identification (e.g., [39,40,41,42,43,44,45,46,47,48]); however none of these works considers the presence of damage in the beam. Moreover, as already mentioned, a change of the axial load cannot be directly related to the presence of a crack in the tie-rod, due to the axial load sensitivity to physical variables, not correlated to the state of health of the tie-rod, and due to environmental effects, e.g., temperature. Only recently, the problem of detecting damage in tie-rods has been faced, with a focus on cracks [17] or corrosion [49,50], and this is an important aspect when SHM of larger structures where tie-rods are in use must be carried out (e.g., [51]). Lucà et al. showed that tie-rod eigenfrequencies can be used as synthetic damage features that are representative of all physical variables which affect the system behaviour, included the axial load. At the same time, they can be used for MSD-based damage detection, when a long-term baseline set is available [18]. However, as mentioned, there are cases when short time baseline is needed.

The novel approach presented in this paper represents a solution to this kind of problems since it adopts a technique allowing for filtering out the temperature effects from the damage index which thus results effective, even in presence of an incomplete set of environmental conditions. This is done by relying on the PCA, which is a well known multivariate analysis technique, often adopted in data representation or data compression [52]. This tool allows projecting the original data set into a new space, defined by the principal components (PCs). The PCs are new variables that are sorted such that the majority of the variability in the original data set is explained by the first few PCs. Since under normal operational conditions the majority of the variability of a multivariate damage feature set is due to environmental effects, it is reasonable to expect that the first few PCs will be representative of these effects [19,34,53]. The idea behind the damage detection algorithm developed in this work is to exclude these PCs and, then, to use the remaining ones to define a damage index which is, thus, insensitive to environmental effects. To show the effectiveness and the reliability of this novel PCA-based procedure, it will be compared with one of the most used approaches in this field, which is the MSD-based method presented in [18]. The comparison will be carried out both on simulated and experimental data of axially-loaded beams.

The article is organized as it follows: in Section 2, both the MSD-based and the PCA-based damage detection algorithms are explained. Moreover, the simulated data and the experimental set-up are described. In Section 3, the results of the simulations are showed and discussed. The experimental results are presented and commented in Section 4. Finally, the conclusions are drawn in Section 5.

## 2. The New PCA-Based SHM Approach and the Validation Plan

In this section, the two methods that are compared in this paper are introduced. Furthermore, a description of the simulated and experimental data is provided.

Before entering into details of the two compared approaches, it is worth mentioning that the initial damage feature is a collection of eigenfrequencies of the monitored tie-rod. This starting point comes from previous research works where it has been proved that the eigenfrequencies of an axially-loaded beam-like structure, used as a multivariate damage feature, can be effectively adopted to spot damage in operating tie-rods [18,49,50,54].

Indeed, eigenfrequencies can be used to synthetically represent the state of the monitored tie-rod, since they are representative of the physical variables that mostly influence its dynamic behaviour (e.g., the axial load). Moreover, the effect of environmental changes is different from that of damage, if multiple eigenfrequencies are considered as a multivariate damage feature. As an example, the eigenfrequencies of the first four bending vertical modes of a healthy tie-rod are considered: a decrease of temperature would cause an increase in the values of all four eigenfrequencies and the lower the vibration mode considered, the higher the effect [18]. If, instead, the temperature does not change but damage (e.g., a reduction of flexural stiffness) is present at midspan, only the eigenfrequencies of the first and third vibration modes would change, since midspan is a vibration node for the even vibration modes. Furthermore, the higher the vibration mode considered, the higher the effect [18].

If a number *m* of vibration modes are considered, the associated eigenfrequency values are referred to as $f_{1},f_{2},\cdots ,f_{i},\cdots ,f_{m}$, with i=1,2,⋯,m (according to this notation, the eigenfrequencies are sorted in ascending order and i=1 simply indicates the lowest eigenfrequency value among those considered, not necessarily that associated to the first vibration mode). The eigenfrequency values can be arranged in a column vector f, defined as it follows:

(1) $$f={\left[f_{1},f_{2},\cdots ,f_{i},\cdots ,f_{m}\right]}^{T}$$

where the superscript “${}^{T}$” means the transpose.

The vector f constitutes the damage feature and it is used to represent the state of the structure with few variables, with respect to the raw acceleration data. When the structure is monitored over time, the feature vector can be estimated several times. In this case, a generic number *r* of feature vectors $f_{1},f_{2},\cdots ,f_{j},\cdots ,f_{r}$, with j=1,2,⋯,r can be arranged in a matrix F as it follows:

(2) $$F=\left[\begin{array}{c} f_{1}^{T}\\ f_{2}^{T}\\ \vdots \\ f_{j}^{T}\\ \vdots \\ f_{r}^{T} \end{array}\right].$$

In the following, the symbol $F^{0}$ will be adopted to indicate the baseline set, i.e., a matrix containing a number *b* of observations (i.e., r=b) of the damage feature when the structure is in the reference initial condition and which will be used for training the methods. The symbol $f^{*}$ will be adopted to indicate a generic new observation of the damage feature which does not belong to the baseline set, thus, it is associated to an unknown health condition. Finally, the symbol $F^{*}$ will be adopted to indicate a set containing a number *n* of observations of the damage feature (i.e., r=n) that do not belong to the baseline set, thus, $F^{*}$ can potentially include damage-related data.

### 2.1. The Benchmark MSD-Based Approach

In this paper, the benchmark is represented by an MSD-based damage index. The MSD is a well-known multivariate metrics, often adopted in the field of SHM to define damage indexes. In the considered case, the MSD between the new vector $f^{*}$ and the baseline set $F^{0}$ can be evaluated according to the following expression:

(3) $$d^{\mathrm{MSD}}={\left(f^{*}-\mu^{0}\right)}^{T}{\left(\Sigma^{0}\right)}^{-1}\left(f^{*}-\mu^{0}\right)=MSD\left(f^{*},F^{0}\right)$$

where $\mu^{0}$ is a m×1 vector where every *i*-th element is the mean of the *i*-th column of $F^{0}$, $\Sigma^{0}$ is the covariance matrix of $F^{0}$ and “${}^{-1}$” means the inverse. The notation $MSD(f^{*},F^{0})$ is used here to indicate the result of the application of the MSD operator to the vector $f^{*}$ with respect to $F^{0}$. It is also noticed that the equivalent vector operator $MSD(F^{*},F^{0})$ used further in the paper indicates the MSD operator applied to each observation contained in $F^{*}$ with respect to $F^{0}$, resulting in a n×1 vector. The result of Equation (3), the MSD, is a scalar number and constitutes the damage feature of the benchmark method.

To detect possible structural changes, the scalar value $d^{\mathrm{MSD}}$ has to be checked against a threshold to state whether the vector $f^{*}$ can be considered as an outlier with respect to the set $F^{0}$. The threshold can be set according to a procedure based on the Monte Carlo method explained in [2,55], briefly described in the following:

- construct a matrix of size b×m, where every element is a random number generated from a zero mean and unit standard deviation normal distribution;
- calculate the MSD between the transpose of each row of the matrix and the the matrix itself;
- store the maximum of the *b* obtained distances;
- repeat the operation for a large number of trials, e.g., 1000, and then sort all the maxima in terms of magnitude;
- the inclusive threshold *t* is then defined as the 95th percentile of the distribution of the MSD maxima (the term inclusive refers to a case when the baseline may also contain damaged or altered data which will be, thus, considered as outliers);
- if the baseline set does not include outliers, the exclusive threshold $t^{\mathrm{MSD}}$ must be adopted. The threshold $t^{\mathrm{MSD}}$ can be calculated according to the following equation:
  (4) $$t^{\mathrm{MSD}}=\frac{\left(b-1\right){\left(b+1\right)}^{2}t}{b\left(b^{2}-\left(b+1\right)t\right)}.$$

**Figure 1:**
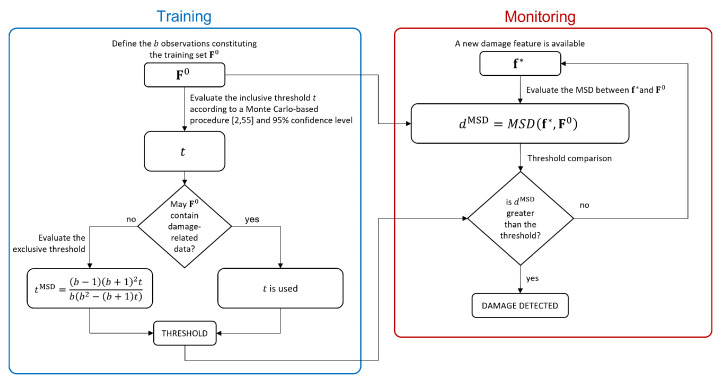
Flowchart of the MSD-based approach.

Summarizing, the main steps required by the MSD-approach used as a benchmark in this work are shown in the flowchart reported in Figure 1.

The MSD is very popular in the field of SHM since this metric naturally filters out the variability associated with the environmental effects while keeping a high sensitivity to structural changes [35]. However, it is known that to properly filter out environmental effects, a full range of environmental conditions must be included in the baseline set to describe the whole variability of the considered feature in operational conditions. In real applications, which are usually characterized not only by short-term trends but also seasonal ones, this aspect translates in the need for long baseline sets. In the following section, a new approach is proposed to try to overcome this limit.

### 2.2. The PCA-Based Approach

The new proposed approach is obtained through the adoption of the PCA. The PCA is a multivariate analysis technique that allows an orthogonal projection of a given data set onto a different coordinate system, where each of the new coordinates (the PCs) accounts for a decreasing amount of the variance of the original data set. The PCs are uncorrelated each other and they are sorted so that the first few components retain most of the variability present in the original data set.

This new description of the data set is usually adopted when a dimensionality reduction is needed. Considering just the very first PCs allows, indeed, to retain most part of the data set variability with a few number of variables. Here, instead, PCA is used for a different purpose. Its aim will be the removal of the data variability due to operating and environmental changes, as will be clarified in the following.

In this case, the data set $F^{0}$ (of size b×m) must be centred by subtracting the mean of each column from each value in that column, obtaining the matrix $C^{0}$. Then, the PCA transforms the data in $C^{0}$ into a new set $Z^{0}$ (the score matrix) through a rotational transformation according to the following equation:

(5) $$Z^{0}=C^{0}R$$

where R is an m×m matrix (the loading matrix). The matrix $Z^{0}$ contains the scores in the principal directions of $C^{0}$, arranged such as the first column contains the scores related to the PC accounting for the largest variance, the second column contains the scores related to the PC accounting for the second largest variance, and so on. The matrix R can be evaluated, for example, by adopting the singular value decomposition. The reader can refer to [52] for a complete theory on the topic.

In the proposed framework, the PCA is used to project the centred baseline matrix $C^{0}$ and obtain the scores associated to the PCs $Z^{0}$. As it will be shown in the following sections, in the baseline data set, where no damage occurs (i.e., the baseline data set is considered as the reference structural condition), the majority of the variance in the eigenfrequencies is associated with temperature effects. The idea is, then, to remove the first *p* columns of the matrix $Z^{0}$, associated to the first *p* PCs, to filter out the temperature effect from the baseline dataset. Once the first *p* columns of the matrix $Z^{0}$ are removed, the matrix ${\hat{Z}}^{0}$ is obtained (in the following, the hat symbol is used to indicate score matrices after the removal of the first *p* columns).

The key idea of the new SHM procedure proposed here is that, when new observations of the feature vector are available, if they are still referring to the same structural condition as the baseline, the PCA should project the data in the same principal directions (i.e., the transformation matrix R is still the same). Let’s consider the matrix $F^{*}$, containing *n* new feature vectors $f^{*}$ which are not included in the baseline. A matrix $F^{0*}$ can be assembled as it follows:

(6) $$F^{0*}=\left[\begin{array}{c} F^{0}\\ F^{*} \end{array}\right].$$

Following the same steps previously described for $F^{0}$, the matrix $F^{0*}$ is centred and the PCA is applied obtaining $Z^{0*}$. Then, the first *p* columns are, again, removed from the score matrix, obtaining the matrix ${\hat{Z}}^{0*}$.

Now, the MSD is calculated between each element of ${\hat{Z}}^{0*}$ and ${\hat{Z}}^{0}$, i.e.,:

(7) $$d=MSD({\hat{Z}}^{0*},{\hat{Z}}^{0})$$

and the result is a vector d, containing the MSD of the transpose of each row of ${\hat{Z}}^{0*}$ with respect to ${\hat{Z}}^{0}$.

The vector d is a $\left(b+n\right)\times 1$ column vector. The first *b* distances contained in d are considered, and the number *o* of these *b* distances which exceed a reference value (further indicated as $P_{0.95}$, see below) is counted. The new damage index is defined as it follows:

(8) $$d^{\mathrm{PCA}}=\frac{o}{b}.$$

In order to calculate the damage detection threshold $t^{\mathrm{PCA}}$, the procedure described in the following is adopted:

- Only the baseline is considered and the MSDs are calculated between the transpose of each row of ${\hat{Z}}^{0}$ and the matrix ${\hat{Z}}^{0}$, i.e.,:
  (9) $$d^{0}=MSD\left({\hat{Z}}^{0},{\hat{Z}}^{0}\right).$$
- A probability density function is estimated, by fitting a Gamma distribution [56] to the elements in $d^{0}$.
- The 95th percentile $P_{0.95}$ and its lower and upper 95% confidence bounds, $P_{0.95,\mathrm{UB}}$ and $P_{0.95,\mathrm{LB}}$, are extracted.
- The number of the first *b* elements of $d^{0}$ exceeding $P_{0.95}$, $P_{0.95,\mathrm{UB}}$ and $P_{0.95,\mathrm{LB}}$ are counted, obtaining respectively *c*, *u* and *l*.
- *c*, *u* and *l* are then normalized with respect to the number *b* of elements in the baseline, obtaining the damage threshold $t^{\mathrm{PCA}}$ and its 95% confidence bounds, i.e.,:
  (10) $$t^{\mathrm{PCA}}=c/b$$
  (11) $$t^{\mathrm{PCA},\mathrm{up}}=u/b$$
  (12) $$t^{\mathrm{PCA},\mathrm{lo}}=l/b.$$

**Figure 2:**
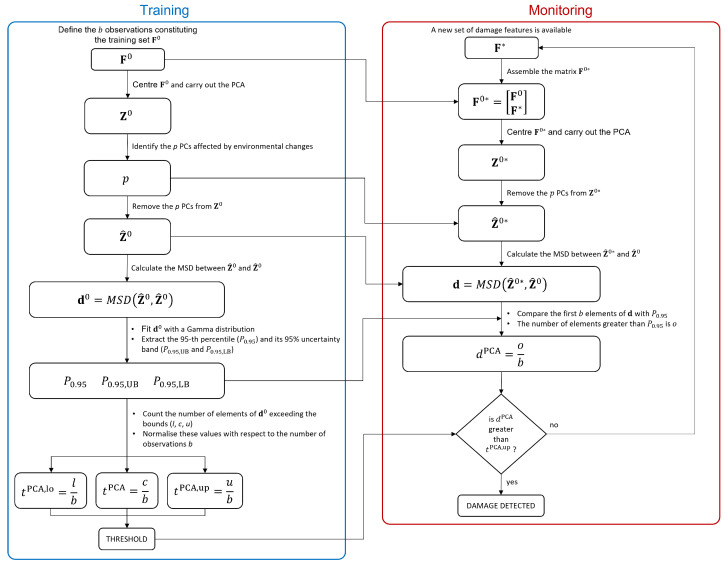
Flowchart of the PCA-based approach.

Finally, the possible presence of a damage is assessed if $d^{\mathrm{PCA}}$ exceeds $t^{\mathrm{PCA},\mathrm{up}}$. This indeed means that more than 5% of the first *b* elements of d exceed the 95th percentile $P_{0.95}$ (with a confidence level of 95%), implying that the new d does not belong to the Gamma distribution fitted on $d^{0}$, thus suggesting the presence of damage. Finally, the main steps required by the proposed PCA-based approach are shown in the flowchart reported in Figure 2.

A difference between the new PCA-based approach and the MSD-based one is that $d^{\mathrm{MSD}}$ compares a single observation $f^{*}$ with the baseline set, while $d^{\mathrm{PCA}}$ requires a set of new samples $F^{*}$ to be assembled with the baseline matrix $F^{0}$. Thus, after the baseline data set, each time a new observation of the damage feature is available, the matrix $F^{0*}$ will be increased of one row, until the number of new observations is equal to *n*. From that moment onward, the matrix $F^{0*}$ will always have size b+n, meaning that, every time a new observation is available, it is included in $F^{*}$ and the least recent one is discarded, proceeding as a travelling window.

The length of the data set $F^{*}$ (i.e., *n*) defines the sensitivity and the readiness of the method in detecting the damage, as will be mentioned later in the paper. Indeed, if *n* is much lower than *b*, and $F^{*}$ contains data referring to an altered condition, their weight in the coordinate transformation of $F^{0*}$ will be low. If *n* is much higher than *b*, when an alteration occurs the method will show the damage effect only when a certain number of damaged samples will replace the undamaged ones in $F^{*}$. This translates in a transient effect and the higher *n* with respect to *b* is, the slower the transient is. In this application we, thus, choose to use n=b as a compromise.

Finally, it should be noted that the steps required by the proposed method during the monitoring phase (see Figure 2) can be carried out through computationally inexpensive numerical algorithms (e.g., the above mentioned singular value decomposition to carry out the PCA). This means that the health condition of the considered structure can be evaluated in near-real time, every time a new observation of the damage feature is available.

### 2.3. PCA-Based Method Validation: Simulations and Experiments

The two methods presented in Section 2.1 and Section 2.2 will be compared using both simulated and experimental data. Two aspects will be investigated: the effectiveness in filtering out the effects of environmental variables and in detecting damage of different severity. To this purpose, different situations were simulated:

1. cases with no damage and with a cyclic (sinusoidal) temperature trend, simulating its daily or seasonal variations;
2. cases with no damage and two cyclic temperature trends, simulating both daily and seasonal variations;
3. cases with damage and two cyclic temperature trends;
4. cases with no damage and temperature trends coming from experimental measurements (i.e., real temperature variations);
5. cases with damage and temperature trends coming from experimental data.

Cases 1 and 2 allow comparing the effectiveness of the two methods in filtering out the temperature effects when considering a whole temperature cycle (i.e., one period of the main sine) or a fraction of it in the training set. Case 3 allows the assessment of both the ability of the methods in filtering out the environmental effects and their sensitivity to damage of different severities. Finally, cases 4 and 5 remove the constraint of pure cyclic trends using real temperature data, thus allowing for an evaluation of the robustness of the methods to generic temperature variations.

Furthermore, again with the same aim of validating the proposed method in different situations and comparing its results with a benchmark SHM method, experimental tests were then performed. The tests were conducted on a sample structure placed in a room with monitored but uncontrolled temperature conditions. Data were acquired both without damage and with a purposely introduced damage with different severity levels, thus allowing for a validation of the simulation results, in terms of method behaviour.

This section will present in detail the simulations carried out and the experimental set-up, while the comparison results will be presented in Section 3 and Section 4.

#### 2.3.1. The Simulations

The simulations are meant to investigate the sensitivity of the two methods to environmental changes and to estimate their effectiveness in separating temperature and damage effects. The case of a simply supported axially-loaded beam is considered, for which the eigenfrequency values for the bending vertical modes can be analytically estimated, according to the following equation [57,58,59]:

(13) $$f_{i}=\frac{i}{2L}\sqrt{\frac{S+EJ(\frac{i^{2}\pi^{2}}{L^{2}})}{q}}.$$

In Equation (13), *L* is the tie-rod length, *S* is the axial load, *E* is the Young’s modulus, *J* is the momentum of inertia of the cross section and *q* is the mass per unit length. The simulations were carried out on a beam with rectangular cross-section with height *h* and width *w*. Thus, $J=(w\;h^{3})/12$ and q=whρ, where ρ is the material density.

Eigenfrequency time-trends are generated by changing the axial load value, with respect to an initial reference value $S_{0}$, which corresponds to a generic initial temperature $T_{0}$. A linear relationship between the axial load and the temperature *T* is assumed, i.e., $S=S_{0}+k\;\left(T-T_{0}\right)$, where *k* is a constant (i.e., the slope of the line that describes the axial load as a function of the temperature). For this reason, in Section 3, temperature trends for simulated data will always be represented as the difference with respect to the initial temperature $T_{0}$, i.e., $T-T_{0}$. The reference values adopted for the simulations are reported in Table 1.

Temperature trends, made by either a single sinusoidal trend or two sinusoidal trends, are simulated. If the latter case is considered, both long-term and short-term cyclic trends are present, to mimic seasonal and daily temperature fluctuations, respectively. A simple sine function with amplitude equal to 8 °C and mean equal to 0 °C is used for the long-term temperature trend, which represents the seasonal trend of the mean daily temperature. A series of sinusoidal functions characterized by a shorter period are used to represent the cyclic daily fluctuations. Each of the short-term sinusoidal functions has mean equal to 0 °C and amplitude which is randomly extracted from uniformly distributed numbers in the interval between 1.5 and 4 °C, to simulate that the thermal excursion may change from day to day. The two trends, i.e., the long-term sine function and the series of short-term trends, are summed up, to obtain the simulated temperature with two cyclic components. Conversely, simulated temperature trends with a single cyclic component are pure sines with amplitude equal to 8 °C, as the seasonal trend described above. The temperature values adopted to define the amplitudes of short-term and long-term trends are similar to those registered by meteorological outdoor stations, located in the north of Italy. Finally, the possibility to simulate eigenfrequency trends as function of the temperature allows also to use real temperature data as an input (see Section 3.4 and Section 3.5). Also in this case, temperature data are represented as variations with respect to a reference mean value.

The effect of damage is, then, introduced as a reduction of Young’s Modulus of a portion of the tie-rod of extent equal to 1% of *L*, at midspan. The way to introduce the effect of damage is by reducing each *i*-th eigenfrequency value, provided by Equation (13), by a certain percentage $\Delta f_{i}$. The values for $\Delta f_{i}$ are obtained through finite element simulations carried out considering a three-dimensional axially-loaded beam model. The reader can refer to [18], where complete details on the finite element simulations are provided. In this work, two different damage levels are considered, i.e., 10% and 30% of Young’s modulus reduction (for both damage conditions, the values for $\Delta f_{i}$ for the first five tie-rod eigenfrequencies are reported in Table 2). A summary of all the simulated test cases is shown in Table 3.

The outcome of the simulations is discussed in Section 3. In the next subsection, the experimental set-up is described.

#### 2.3.2. The Experiments

**Figure 3:**
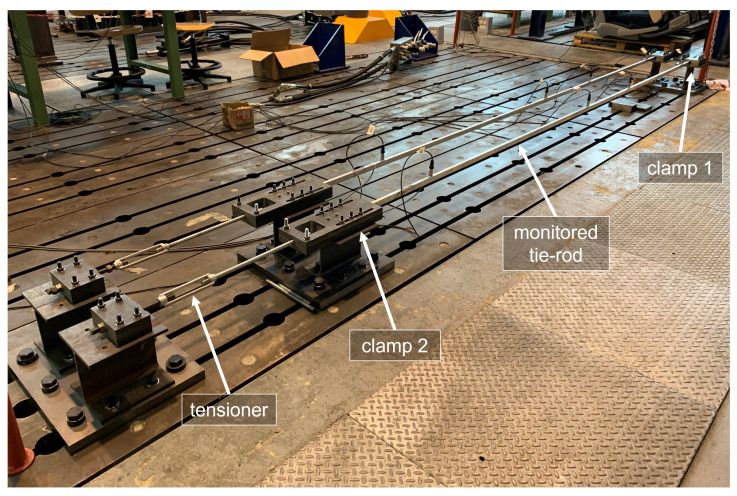
The experimental set-up.

The experimental data come from a test bench (see Figure 3) installed in the Mechanical Engineering laboratory of Politecnico di Milano, in Italy. A full-scale aluminium tie-rod is considered, characterized by a free length of 4 m and a cross-section equal to 0.015×0.025 m^2^.

Clamps made from steel plates are located at the two ends of the beam, to provide the constraints. The plates are in contact with the upper and lower faces of the tie-rod and they are held together by bolted joints. During the installation, the bolted joints of one of the two clamps (clamp 1 in Figure 3) were tightened, while the ones of the other clamp (clamp 2 in Figure 3) were left loose. In this way, since the beam was not fully constrained along the axial direction, a tension was applied through a tensioner. When a tension of 8000 N was applied to the tie-rod, also the bolted joints of clamp 2 were tightened up, to finally obtain a tensioned beam with a clamped-clamped constraint configuration.

Preliminary tests revealed that the broadband excitation provided by the environment, under normal conditions, significantly decreases for frequencies higher than 200 Hz and that the vibration modes which are mostly excited by the operational environment are the first six bending vertical modes (the eigenfrequency values for the first six bending vertical modes, identified through an impact hammer test carried out immediately after the tensioning procedure, are reported in Table 4).

The choices related to the sensor layout were aimed to obtain a sufficient spatial resolution to distinguish the mode shapes associated with the first six bending vertical modes, using as few sensors as possible, in order to reduce the load effect and to mimic real applications. Indeed, the use of as few sensors as possible is often desirable in field applications, for both practical and economic reasons. Many different layouts were evaluated based on the autoMAC matrix [60], to finally select a layout composed of four uniaxial accelerometers, fixed on the top face of the tie-rod, at distances of $\frac{1}{20}L$,$\frac{1}{3}L$,$\frac{1}{2}L$ and $\frac{9}{10}L$ from clamp 1. However, it should be noted that the choice of considering only bending modes in the vertical plane is specific of this experimental campaign. Indeed, by using, e.g., triaxial accelerometers, also other vibration modes, as the bending lateral ones, can be included in the analysis.

More in detail, the adopted accelerometers are general-purpose industrial piezoelectric accelerometers, model PCB603C01 (sensitivity of 10.2 mV/(m/s^2^), full scale of ±490 m/s^2^). The choice for general-purpose industrial accelerometers comes from the decision to not adopt high-end sensors, which are typical of laboratory environments and not representative of real applications. Moreover, axially-loaded beam-like structures are usually subject to significant vibration levels in operational conditions, due to their slenderness, making possible the use of, e.g., industrial piezoelectric accelerometers or accelerometers based on microelectromechanical systems (MEMS). Regarding the acquisition system, it is composed by NI 9234 modules with anti-aliasing filter on board and the sampling frequency is set to 512 Hz, obtaining a bandwidth of approximately 200 Hz that includes the range of frequency significantly excited by the operational environment.

It must be pointed out that neither the temperature nor the excitation are controlled, thus, even though it is a laboratory experiment, acquired data are similar to those of real monitoring applications. The temperature reaches minimum values approximately equal to 5 °C, during winter, and maximum values approximately equal to 30 °C during summer. Daily thermal excursion ranges from ±1.5 °C to ±4 °C.

The characteristics of the operational environment allow for a stable modal identification of the first four bending vertical modes, through the adoption of the polyreference least-square complex frequency-domain method [61]. Thus, the eigenfrequencies used to calculate the damage indexes in Section 4 are those of the first four bending vertical modes. However, the proposed strategy is of general validity and can also be used when other output-only modal identification algorithms are adopted to extract the required number of modal parameters. Furthermore, since only the eigenfrequency values are used to calculate either the MSD-based or the PCA-based damage indexes, also the adoption of a single accelerometer and simple single-degree-of-freedom output-only techniques is possible [50].

The damage effect has been introduced through the addition of a concentrated mass, to alter the dynamic properties of the tie-rod with a simple and reversible strategy, often used in literature (e.g., in [62,63,64,65]). Damage is simulated close to the constraints, at a distance equal to $\frac{1}{10}L$, which represents a challenging scenario for eigenfrequency-based damage detection [18]. Two different masses are used, equal to 1% and 3% of the total mass of the beam, to test different damage severity.

## 3. Results: Simulations

In this section, the results of the simulations are presented. The different subsections discuss the results of the simulations 1 and 2, 3, 4, 5 and 6, 7 and 8, respectively, described in Table 3 and associated to different temperature and damage conditions.

### 3.1. Long-Term Temperature Trends and No Damage

**Figure 4:**
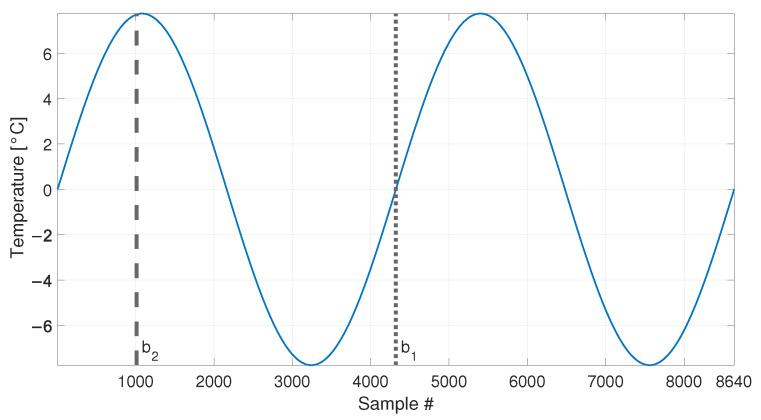
Simulated temperature trend: long-term trend only. Vertical dotted and dashed lines identify the number of samples used as baseline in sim 1 and sim 2, respectively.

At first, the temperature profile reported in Figure 4 is considered, which is composed by 8640 samples. In this set of simulated data, the eigenfrequency changes are only associated to the temperature change and the tie-rod is always in the same healthy condition.

**Figure 5:**
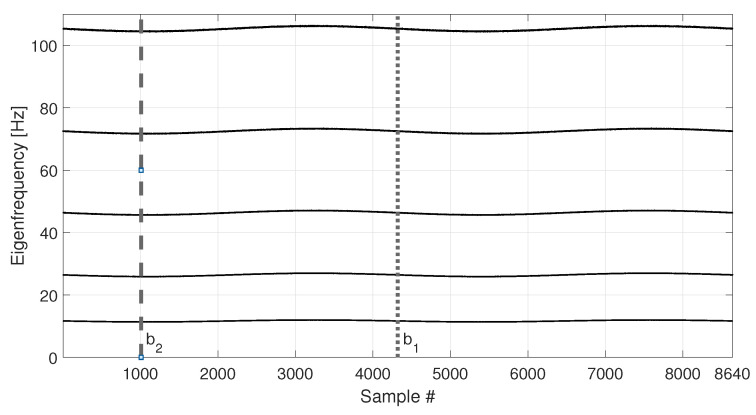
Simulated eigenfrequency trends due to long-term temperature trend. Vertical dotted and dashed lines identify the number of samples used as baseline in sim 1 and sim 2, respectively.

The eigenfrequency trends for the first five vertical bending modes of the simulated tie-rod are reported in Figure 5.

The temperature follows a simple sine function and it completes two identical cycles, covering the range −8 to +8 °C with respect to the initial temperature value. As it is reasonable to expect, also the eigenfrequency trends follow the cyclical trend of temperature.

In order to compare the MSD-based and the PCA-based strategies in their capability to filter out the environmental effects, first, a number $b_{1}=4320$ of observations of the damage feature are considered (see Table 3, sim 1), i.e., half of the total number of samples shown in Figure 5 (the limit of the baseline set is represented as a vertical dotted line in Figure 4 and Figure 5). In this way, the baseline set $F^{0}$ includes data referring to an entire temperature cycle, i.e., all the environmental conditions to which the tie-rod is subject.

**Figure 6:**
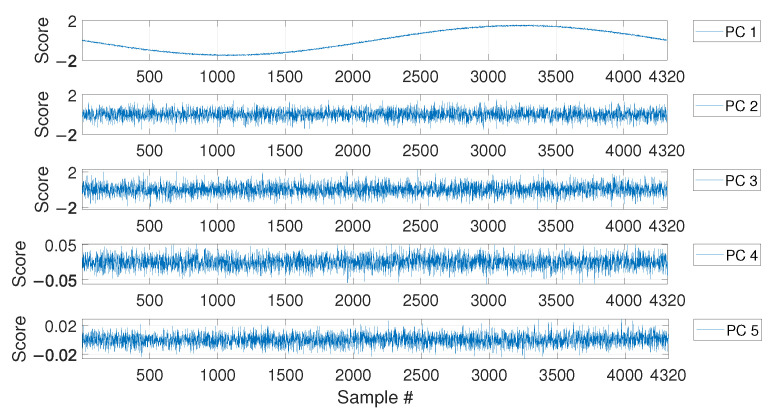
PC scores for the baseline set of eigenfrequencies containing a number b=4320 of observations, considering a long-term temperature trend.

For MSD-based strategy, the damage index $d^{\mathrm{MSD}}$ is evaluated by calculating the MSD of each observation subsequent to the baseline (i.e., samples after $b_{1}$) and compared with the threshold $t^{\mathrm{MSD}}$. As for the PCA-based strategy, Figure 6 shows the PC scores for the baseline set of eigenfrequencies $F^{0}$, i.e., the columns of matrix Z (see Section 2.2). As it is possible to see, the scores in the first principal direction show a deterministic trend that is strictly related to the temperature trend (compare the first plot of Figure 6 with that of Figure 4). Conversely, the scores in the other principal directions do not show deterministic trends. Since the first PC seems to be highly correlated with temperature, it is removed from the damage feature, before the evaluation of $d^{\mathrm{PCA}}$ (p=1, according to Section 2.2).

**Figure 7:**
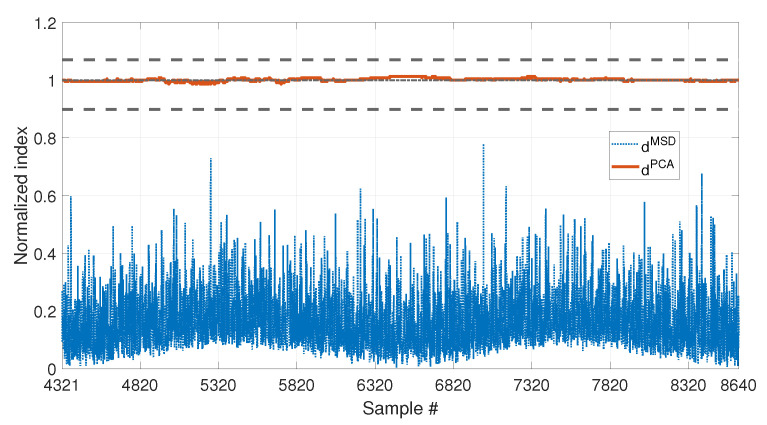
Comparison between $d^{\mathrm{MSD}}$ (blue dotted line) and $d^{\mathrm{PCA}}$ (red solid line), with b=4320, considering a long-term temperature trend.

Figure 7 shows the comparison of the two approaches on the data which are not included in the baseline (i.e., from sample 4321 to sample number 8640). To allow for a direct comparison of the two approaches, from now on, the two indexes $d^{\mathrm{MSD}}$ (blue dotted line) and $d^{\mathrm{PCA}}$ (red solid line) will always be plotted as normalized on the respective damage detection threshold ($t^{\mathrm{MSD}}$ and $t^{\mathrm{PCA}}$, respectively). For this reason, the damage detection threshold is represented by a black horizontal dot-dashed line of value 1 (from now on, referred to as the unitary threshold) for both the methods. In the same way, the upper and lower bounds for the PCA-based threshold (see Equations (11) and 12), $t^{\mathrm{PCA},\mathrm{up}}$ and $t^{\mathrm{PCA},\mathrm{lo}}$, respectively, will be presented as normalized on the damage detection threshold $t^{\mathrm{PCA}}$ and indicated by black horizontal dashed lines.

As it is possible to see by observing the results presented in Figure 7, both the strategies are effective in filtering out the temperature effect. Indeed, $d^{\mathrm{MSD}}$ is below the damage detection threshold and $d^{\mathrm{PCA}}$ is inside the range defined by $t^{\mathrm{PCA},\mathrm{up}}$ and $t^{\mathrm{PCA},\mathrm{lo}}$. Thus, no false positives are detected due to the environmental effects, which are correctly filtered out since all the temperature conditions from sample 4321 to 8640 were included in the baseline set.

The second case discussed considers a shorter baseline set. In this case, the baseline includes a number $b_{2}=1008$ of samples (see Table 3, sim 2), which is approximately one quarter of the entire temperature cycle (see the black vertical dashed lines in Figure 4 and Figure 5, which indicate the end of the baseline set). In more detail, in this case $F^{0}$ contains only the eigenfrequencies associated to temperatures in the range 0 to +8 °C.

**Figure 8:**
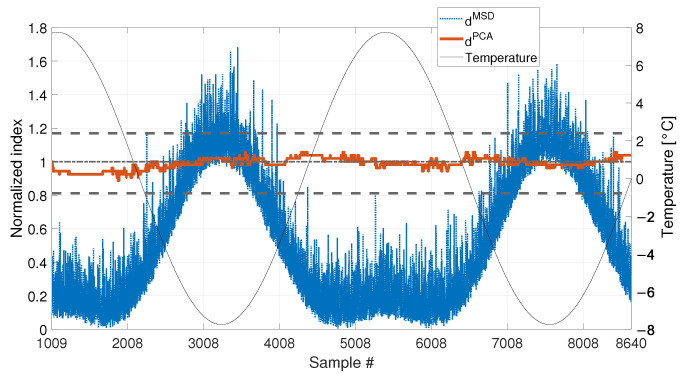
Comparison between $d^{\mathrm{MSD}}$ (blue dotted line) and $d^{\mathrm{PCA}}$ (red solid line), with b=1008, considering a long-term temperature trend (black thin line).

Figure 8 shows the comparison of the two approaches. In this case, also the temperature is plotted on the right axis of the figure with a black thin line, to facilitate the interpretation of the results. As it is possible to see, the PCA-based strategy is still filtering out the temperature effect correctly. Indeed, $d^{\mathrm{PCA}}$ is always inside the range defined by $t^{\mathrm{PCA},\mathrm{up}}$ and $t^{\mathrm{PCA},\mathrm{lo}}$. This confirms that most of the variability of the data, which is associated to temperature, is explained by the first PC. Therefore, removing the first PC from the damage feature allows to filter out any change due to temperature effects. On the contrary, $d^{\mathrm{MSD}}$ clearly shows a deterministic trend, with values that increase when data outside of the training set are considered. This can be stated by observing that $d^{\mathrm{MSD}}$ increases when the temperature is in the range 0 to −8 °C, which is not included in the baseline (e.g., compare $d^{\mathrm{MSD}}$ and the temperature trend from sample 7008 to sample 8008 in Figure 8). The influence of temperature causes the index $d^{\mathrm{MSD}}$ to exceed the damage detection threshold even if no damage is present, thus producing false positives.

### 3.2. Short-Term and Long-Term Temperature Trends with No Damage

The second set of simulations considers a different temperature profile, characterized by two cyclical trends: a long-term trend (which is the same as the first set of simulations) and a short-term trend. This data set is meant to mimic the presence of both seasonal and daily temperature trends. Indeed, the long-term trend again covers a range of temperature from −8 to +8 °C in 4320 samples, and it simulates the seasonal trend of the daily mean temperature. The short-term trend, instead, completes an entire cycle in 144 samples. For every daily cycle, the range of temperatures around the daily mean temperature is generated as described in Section 2.3.1.

**Figure 9:**
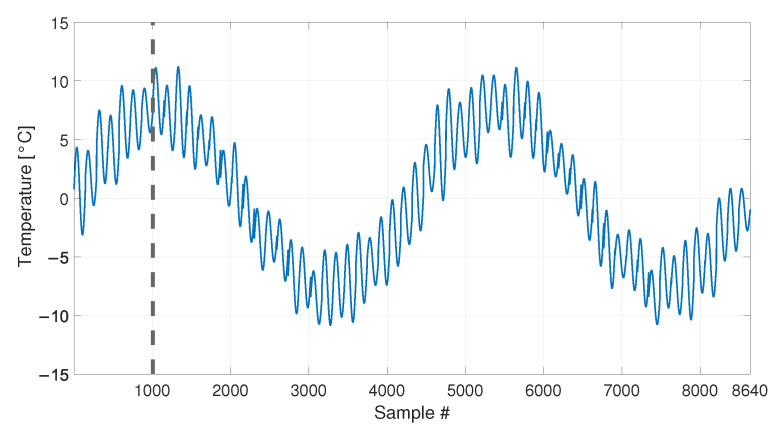
Simulated temperature trend: long-term and short-term trends. The vertical dashed line identifies the number of samples used as baseline in sim 3.

**Figure 10:**
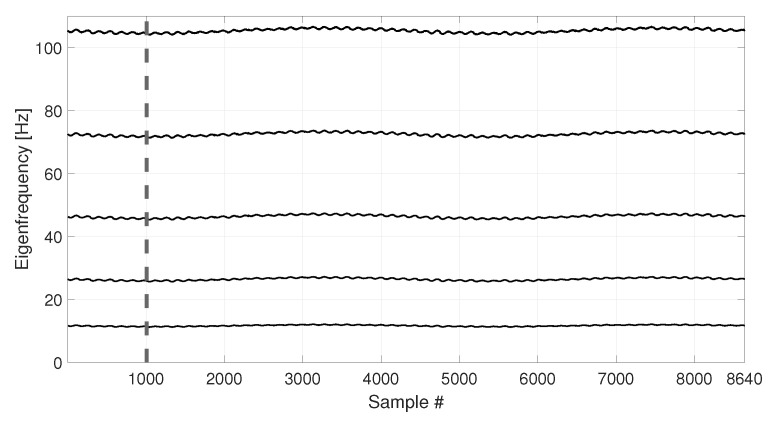
Simulated eigenfrequency trends related to long-term and short-term temperature trends. The vertical dashed line identifies the number of samples used as baseline in sim 3.

The temperature trend shown in Figure 9 is used to simulate the eigenfrequency trends which are reported in Figure 10. As it is expected, also the eigenfrequency trends show both daily and long term trends.

Also in this case, the first $b_{2}=1008$ samples are used as a baseline (see Table 3, sim 3), as indicated by a black vertical dashed line, both in Figure 9 and Figure 10.

**Figure 11:**
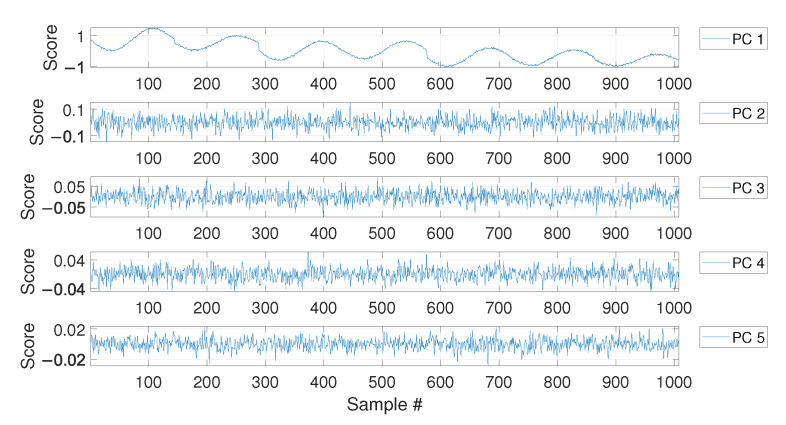
PC scores for the baseline set of eigenfrequencies containing a number b=1008 of observations, considering both long-term and short-term temperature trends.

As in the case of the first set of simulations, the PCA confirms that the first PC has a clear deterministic trend which is highly correlated with the temperature (compare the plot labelled as PC 1 in Figure 11 with the first 1008 samples in Figure 9). Thus, also in this case, the first PC is removed before calculating $d^{\mathrm{PCA}}$.

**Figure 12:**
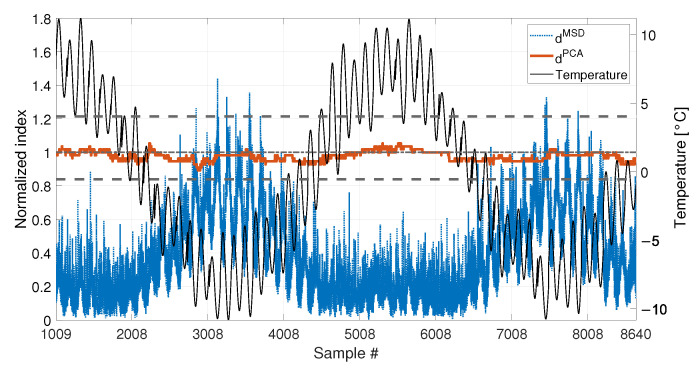
Comparison between $d^{\mathrm{MSD}}$ (blue dotted line) and $d^{\mathrm{PCA}}$ (red solid line), with b=1008, considering both long-term and short-term temperature trends (black thin line).

The comparison of the two approaches is reported in Figure 12 and it shows results which are similar to those commented in Figure 8. The PCA-based strategy is able to filter out the effects of temperature, also in presence of both short-term and long-term temperature trends. The index $d^{\mathrm{PCA}}$ is always in the range defined by $t^{\mathrm{PCA},\mathrm{up}}$ and $t^{\mathrm{PCA},\mathrm{lo}}$. The MSD-based index, instead, shows the same deterministic trend observed in Figure 8, i.e., it increases when the temperature ranges from 0 °C to −8 °C, thus exceeding the damage detection threshold. Moreover, it is possible to notice that also a short-term trend is present in the damage index, which has the same periodicity of the short-term trends in temperature (e.g., compare $d^{\mathrm{MSD}}$ and the temperature trend between samples 3008 and 4008, in Figure 12).

The outcome of these first simulations (sim 1, 2 and 3 of Table 3), where the effect of damage is not accounted for, is that both the strategies are potentially able to be insensitive to temperature effects in the data. However, a strong difference emerged: while the MSD-based strategy requires a complete set of environmental effects to filter them out, the PCA-based strategy can provide a temperature-insensitive damage index without needing for a complete set of environmental conditions. This aspect is relevant in situations where a short baseline set is available, e.g., at the beginning of a monitoring campaign.

### 3.3. Short-Term and Long-Term Temperature Trends with Simulated Damage

This set of simulations aims at answering a central question: are the damage indexes insensitive enough to temperature to allow for damage detection? The simulations discussed in the following, thus, consider the presence of damage.

As mentioned in Section 2.3.1, damage is simulated as a reduction of Young’s modulus of a portion of the tie-rod of extent equal to 1% of the free-length. The portion of the tie-rod which is affected by damage is located at mid-span and two levels of damage are considered: 10% and 30% of Young’s modulus reduction. In order to simulate damage, a change of eigenfrequency value is introduced, using the corresponding eigenfrequency decrease $\Delta f_{i}$ (see Section 2.3.1).

**Figure 13:**
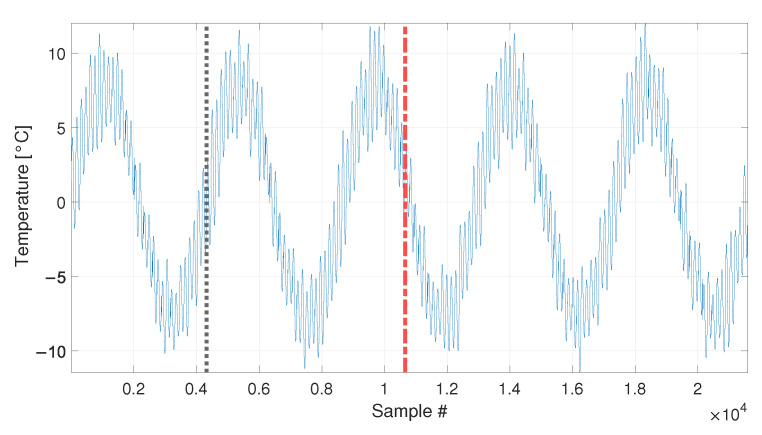
Simulated temperature trend: long-term and short-term temperature trends. The vertical dotted line identifies the number of samples used as baseline in sim 4. The beginning of the damage-related data is indicated by a red vertical dot-dashed line.

In this case, the total number of samples is equal to 21600, which includes five entire long-term temperature trends (see Figure 13). A number equal to $b_{1}=4320$ samples (see Table 3, sim 4) is used to define the baseline, in order to include a complete long-term temperature trend (see the black vertical dotted line in Figure 13).

Damage is introduced after two and a half temperature cycles and the beginning of the sample set containing damage-related data is indicated by a red vertical dot-dashed line in Figure 13.

**Figure 14:**
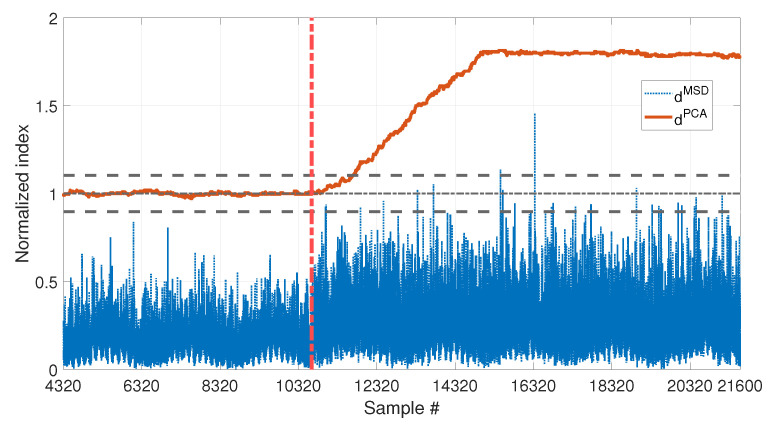
Comparison between $d^{\mathrm{MSD}}$ (blue dotted line) and $d^{\mathrm{PCA}}$ (red solid line), with b=4320, considering long and short-term temperature trends and damage (10% reduction of Young’s modulus).

**Figure 15:**
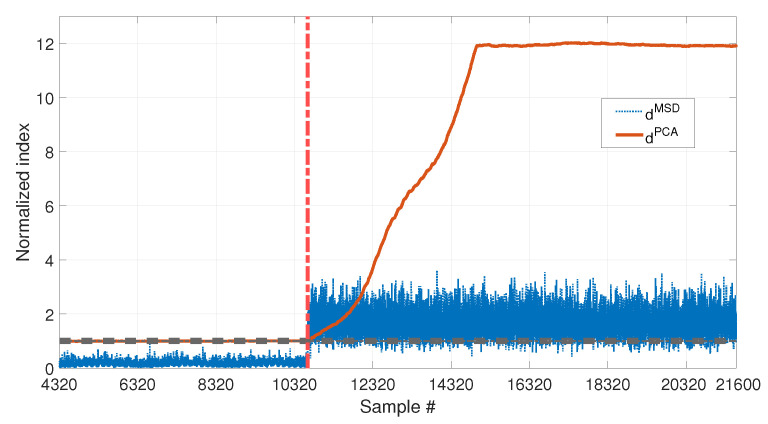
Comparison between $d^{\mathrm{MSD}}$ (blue dotted line) and $d^{\mathrm{PCA}}$ (red solid line), with b=4320, considering long and short-term temperature trends and damage (30% reduction of Young’s modulus).

The performances of $d^{\mathrm{MSD}}$ and $d^{\mathrm{PCA}}$ in presence of damage can be compared, for the two damage levels 10% and 30%, in Figure 14 and Figure 15, respectively.

In both cases, the two indexes are below the respective threshold, when the samples before the beginning of damage are considered, thus they are not producing false positives due to temperature fluctuations (same conclusions of Section 3.1 and Section 3.2). However, the two indexes perform differently when damage occurs: $d^{\mathrm{PCA}}$ is always able to detect damage, exceeding the upper limit of the range defined by $t^{\mathrm{PCA},\mathrm{up}}$ and $t^{\mathrm{PCA},\mathrm{lo}}$, both with low and high damage severity. Moreover it is sensitive to different levels of damage, as proved by the higher level reached by $d^{\mathrm{PCA}}$ in Figure 15 (around 12) than in Figure 14 (around 1.75). In both cases, a transient can be observed that finishes approximately $b_{1}$ samples after the beginning of damage. This is because, due to the travelling window used to calculate $d^{\mathrm{PCA}}$ (see Section 2.2), for the first $b_{1}$ samples after the introduction of damage, $F^{*}$ still contains data referring to the healthy structure.

As for $d^{\mathrm{MSD}}$, the MSD-based damage index is not able to detect the lowest simulated damage, as proved by the fact that $d^{\mathrm{MSD}}$ stays below the unitary threshold in Figure 14. Only the most severe simulated damage is detected ($d^{\mathrm{MSD}}$ almost always above the unitary threshold in Figure 15). However, the conclusion is less clear, if compared with the index $d^{\mathrm{PCA}}$, on the same conditions (compare the blue dotted line with the red-solid line in Figure 15).

Results proved that, when the PCA-based strategy is used, removing the first principal component filters out the temperature effect, while preserving sensitivity to damage. Moreover, $d^{\mathrm{PCA}}$ has a higher sensitivity than $d^{\mathrm{MSD}}$.

### 3.4. Real Temperature Trends without Damage

Before moving to the experimental results, a last set of simulations is discussed. In this case, temperatures are not numerically defined but real temperature values are used. In more detail, the temperature trend comes from the experimental data, collected by a thermocouple in the laboratory where the experimental set-up, described in Section 2.3.2, is located. This set of simulations is meant to check the conclusions of previous Section 3.1–Section 3.3, where simple temperature trends were adopted, to easily separate the effects.

**Figure 16:**
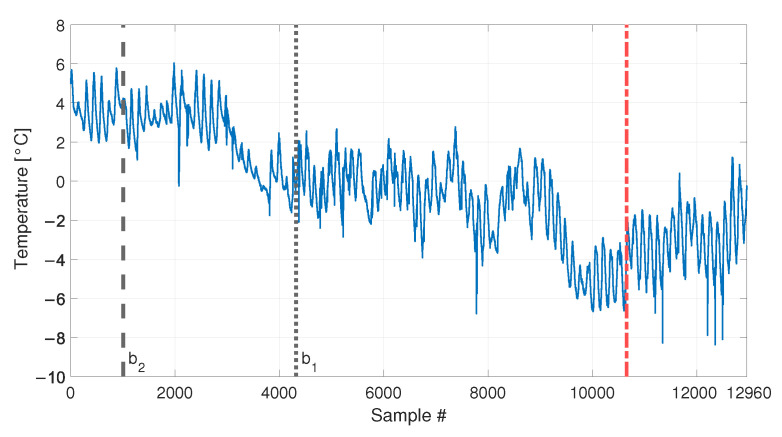
Real temperature trend, including daily trends and long-term drift. Black vertical dotted and dashed lines identify the number of samples used as baseline in sim 6 and sim 5, respectively. The beginning of the damage related data of sim 7 and 8 of Table 3 is indicated by a red vertical dot-dashed line.

The temperature trend used is presented in Figure 16 and refers to the acquisition of the temperature every ten minutes, for a total number of samples equal to 12960 (90 days of data). Data are presented as the difference with respect to the average temperature value. The temperature shows both short-term and long-term trends. The short-term trends show a cyclical behaviour and they are related to the daily temperature trends. Moreover, it is possible to see that the mean daily temperature drifts during the observation window, from values approximately around +4 °C to values approximately around −4 °C.

Two different baselines will be adopted in the following: a short baseline, containing $b_{2}=1008$ samples, see Table 3, sim 5, (the end of the short baseline is indicated by a black vertical dashed line in Figure 16), and a long baseline, containing $b_{1}=4320$ samples, see Table 3, sim 6 (the end of the long baseline is indicated by a black vertical dotted line in Figure 16). As opposite to the previous simulations, it must be noted that even when the longest baseline is considered, it is not enough to include all the temperature values that characterize the remaining part of data. Indeed, the long baseline will include only temperature higher than, approximately, −2 °C, while, in the remaining part of the data, the temperature reaches lower levels.

**Figure 17:**
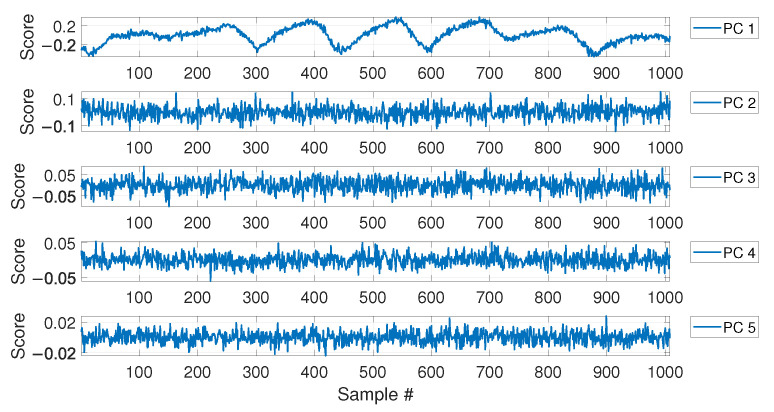
PC scores for the baseline set of eigenfrequencies containing a number b=1008 of observations, considering real temperature trends.

The results of the PCA of the baseline matrix $F^{0}$ again confirmed that the first PC is that presenting a deterministic trend which is highly correlated with that of temperature (see Figure 17). For this reason, again the index $d^{\mathrm{PCA}}$ is calculated after removing the first principal component.

**Figure 18:**
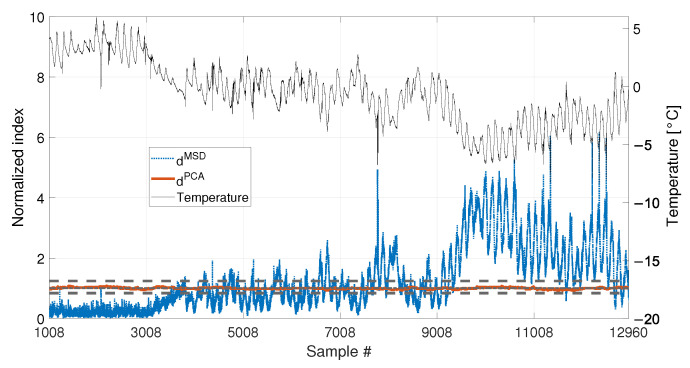
Comparison between $d^{\mathrm{MSD}}$ (blue dotted line) and $d^{\mathrm{PCA}}$ (red solid line), with b=1008, in case of a real temperature trend (black thin line).

**Figure 19:**
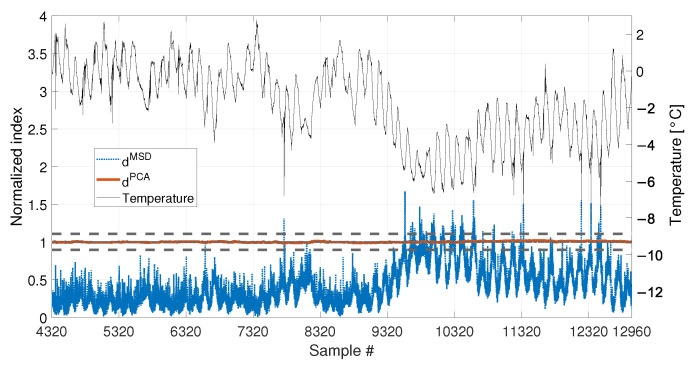
Comparison between $d^{\mathrm{MSD}}$ (blue dotted line) and $d^{\mathrm{PCA}}$ (red solid line), with b=4320, in case of a real temperature trend (black thin line).

Cases where no damage is present are here discussed. The results are presented in Figure 18 for sim 5 (short baseline), and in Figure 19 for sim 6 (long baseline).

With respect to the results presented in Section 3.1 and Section 3.2, the insensitivity of $d^{\mathrm{PCA}}$ to temperature is confirmed: when either 1008 or 4320 samples are considered, $d^{\mathrm{PCA}}$ never exceeds the range defined by $t^{\mathrm{PCA},\mathrm{up}}$ and $t^{\mathrm{PCA},\mathrm{lo}}$, i.e., no false positives are produced. Furthermore, also the performances of the MSD-based strategy are confirmed. Indeed, $d^{\mathrm{MSD}}$ significantly exceeds the damage threshold with the baseline containing 1008 samples, causing false positives. As an example, when the mean trend of temperature decreases around sample 9008 (see Figure 18), the mean trend of $d^{\mathrm{MSD}}$ increases and stays constantly above the threshold. In this case, damage would be detected even if the structure is in healthy condition. Toward the end of the observation window, while temperature increases, $d^{\mathrm{MSD}}$ decreases, coming back to threshold level. Moreover, despite the effect is reduced by adopting a larger baseline (see Figure 19), it is still possible to notice that $d^{\mathrm{MSD}}$ sometimes exceeds the threshold and shows cyclic trends due to daily temperature variations.

### 3.5. Real Temperature Trends with Damage

**Figure 20:**
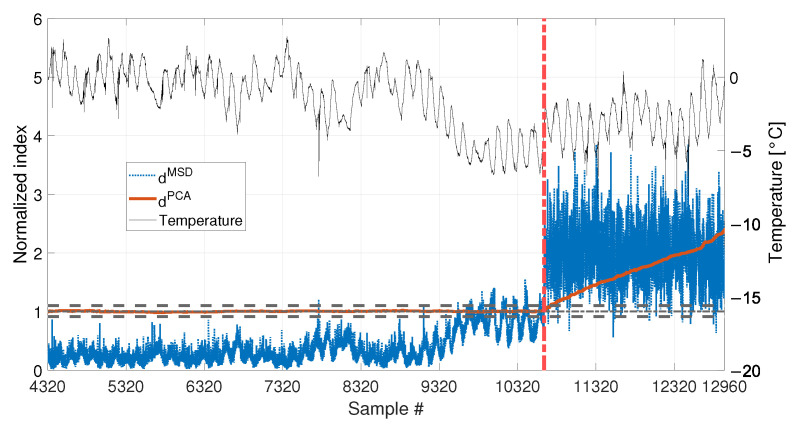
Comparison between $d^{\mathrm{MSD}}$ (blue dotted line) and $d^{\mathrm{PCA}}$ (red solid line), with b=4320, considering a real temperature trend (black thin line) and damage (30% reduction of Young’s modulus).

**Figure 21:**
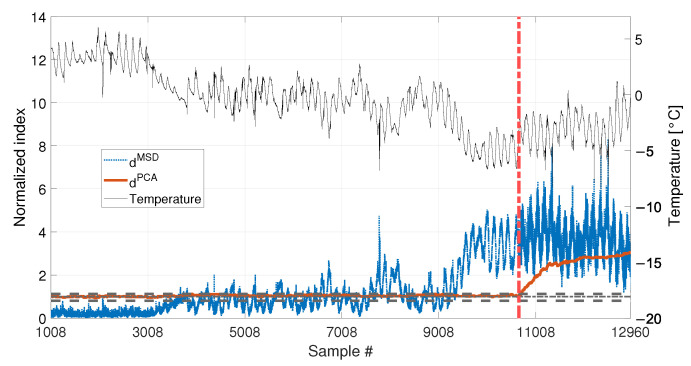
Comparison between $d^{\mathrm{MSD}}$ (blue dotted line) and $d^{\mathrm{PCA}}$ (red solid line), with b=1008, considering a real temperature trend (black thin line) and damage (30% reduction of Young’s modulus).

Finally, the performances in presence of damage are discussed. The results are presented in Figure 20, for the long baseline (see Table 3, sim 7), and in Figure 21, for the short baseline (see Table 3, sim 8). The damage simulated in this case is a 30% reduction of Young’s modulus at midspan and it is indicated by the red vertical dot-dashed line, in Figure 16, Figure 20 and Figure 21.

When a baseline of $b_{1}=4320$ samples are adopted (see Figure 20 and Table 3, sim 7), both strategies are able to detect damage. However, $d^{\mathrm{PCA}}$ shows a clear increasing trend unlike $d^{\mathrm{MSD}}$. The decreasing trend of $d^{\mathrm{MSD}}$ due to temperature, previously observed in Figure 19 (i.e., from about sample 10700 to sample 12960), seems to be mitigated by the effect of damage; however, this effect is still present.

When 1008 samples are adopted (see Figure 21 and Table 3, sim 8) $d^{\mathrm{PCA}}$ is able to clearly detect damage, exceeding the range defined by $t^{\mathrm{PCA},\mathrm{up}}$ and $t^{\mathrm{PCA},\mathrm{lo}}$, confirming that not only the damage index is insensitive to temperature, but it is sensitive to damage. Conversely, the trend of $d^{\mathrm{MSD}}$ is similar to that of Figure 18, where no damage is present. Indeed, damage is detected even when the tie-rod is healthy since $d^{\mathrm{MSD}}$ is above the threshold before damage is introduced (i.e., $d^{\mathrm{MSD}}$ exceeds the threshold approximately at sample 9008). Moreover, the trend of $d^{\mathrm{MSD}}$ immediately before the introduction of damage is similar to that after the introduction of damage. This observation confirms that the increase of $d^{\mathrm{MSD}}$ is mainly due to temperature and not to damage.

## 4. Results: Experiments

In this section, the results obtained by considering real data coming from the experimental set-up (see Section 2.3.2) are presented. Two damage scenarios are considered, where the effect of damage is obtained through the addition of concentrated masses of 1% and 3% of the total mass of the tie-rod, close to one of the two fixed ends.

**Figure 22:**
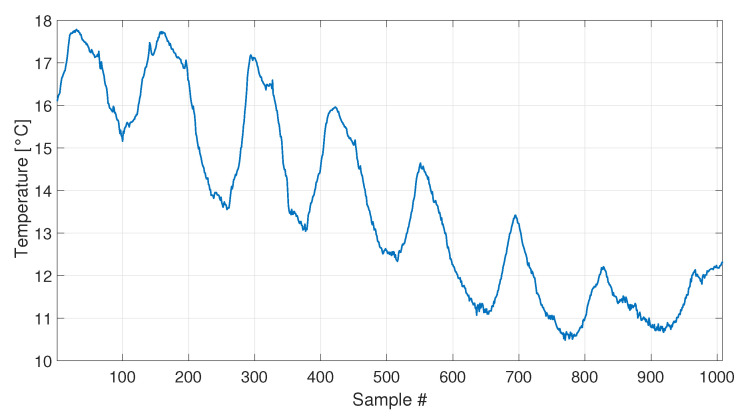
Temperature trend for the baseline set of real data.

**Figure 23:**
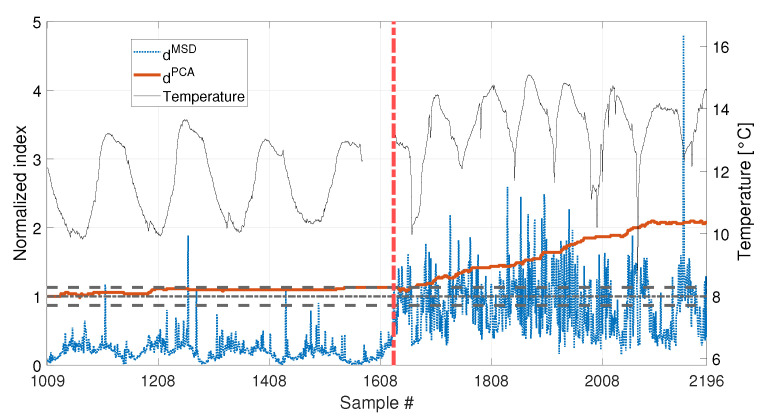
Comparison between $d^{\mathrm{MSD}}$ (blue dotted line) and $d^{\mathrm{PCA}}$ (red solid line), with b=1008, for added mass equal to 1% of the total mass. A black thin line identifies the temperature.

**Figure 24:**
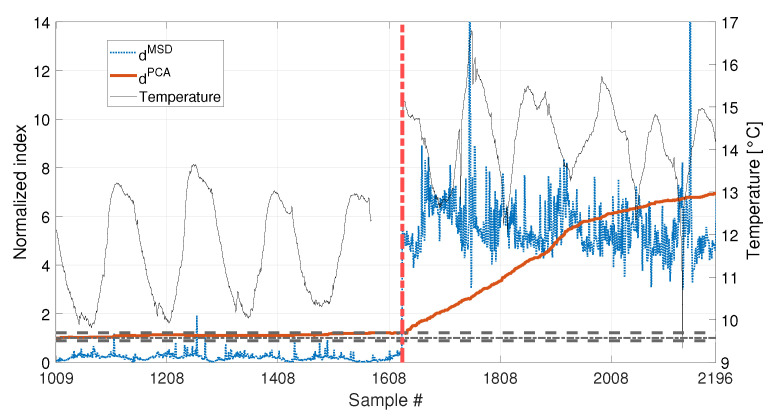
Comparison between $d^{\mathrm{MSD}}$ (blue dotted line) and $d^{\mathrm{PCA}}$ (red solid line), with b=1008, for added mass equal to 3% of the total mass. A black thin line identifies the temperature.

A set of 1008 samples is used to define the baseline matrix $F^{0}$ (i.e., b=1008), composed by the experimentally identified eigenfrequencies for the first four vibration modes (see Section 2.3.2). Considering that an estimate of the four eigenfrequencies is available every 10 min, the baseline set includes 7 days. The temperature trend related to the baseline set is reported in Figure 22, and the temperature trends of the validation and damage sets are reported in the following Figure 23 and Figure 24, together with the damage indexes. It is noticed that, in all the figures related to the experiments, the temperature *T* is plotted in place of $T-T_{0}$. The gap of temperature data noticeable in Figure 23 and Figure 24 is due to missing data caused by a malfunctioning of the temperature sensor. As it is possible to observe, the daily temperature cycles can be clearly noted. Furthermore, a drift in the daily mean temperature is also present. The available baseline set approximately covers the range of temperatures 10.5 to 17.5 °C.

The comparison between the two approaches is presented in Figure 23 and Figure 24, for an added mass of 1% and 3% of the total mass, respectively. The indexes (blue-dotted trend for $d^{\mathrm{MSD}}$ and red-solid trend for $d^{\mathrm{PCA}}$) are normalized on the respective damage detection threshold, as done in the simulations. The horizontal dot-dashed line represents the threshold for $d^{\mathrm{MSD}}$, while the two horizontal dashed lines, below and above the unitary threshold, indicate the range defined by $t^{\mathrm{PCA},\mathrm{up}}$ and $t^{\mathrm{PCA},\mathrm{lo}}$, for $d^{\mathrm{PCA}}$ (see Section 2.2). The right y-axis is used to represent the temperature (black-thin line).

As for the PCA-based strategy, the effects of temperature on the variance of $F^{0}$ is retained by the first two PCs. They indeed show deterministic trends and, thus, were removed from the damage feature. The proposed procedure proved to be able to effectively filter out the temperature effect, as it can be seen from Figure 23 and Figure 24. Indeed, $d^{\mathrm{PCA}}$ does not show any temperature-correlated trend (compare the red and black curves) and, when no damage is present, it does not exceed the range limited by $t^{\mathrm{PCA},\mathrm{up}}$ and $t^{\mathrm{PCA},\mathrm{lo}}$.

In both cases, $d^{\mathrm{PCA}}$ shows a clear growing trend when damage is introduced, thus the PCA-based damage index is able to promptly detect damage. By comparing the trends of $d^{\mathrm{PCA}}$ in Figure 23 and Figure 24, it is possible to observe that the PCA-based damage index is sensitive to different magnitudes of damage: indeed, when damage is 3% the index grows faster than when damage is 1% (compare the values of the red solid trends in Figure 23 with those of Figure 24).

It is worth noticing that only the most severe damage condition (i.e., 3% of added mass) is clearly detected by $d^{\mathrm{MSD}}$. For the case of 1% of added mass, instead, it remains below the threshold for most of the samples and just a slight damage index increase can be deduced, not allowing for a clear damage detection.

The experimental results confirmed what observed on the simulated data: when just few temperature conditions are available to define the baseline data set, the PCA-based strategy can provide a damage index which is robust with respect to the environmental effects, while the MSD-based index is still sensitive to temperature. Moreover, not only $d^{\mathrm{PCA}}$ is less sensitive to temperature than $d^{\mathrm{MSD}}$, but $d^{\mathrm{PCA}}$ has a higher sensitivity to damage than $d^{\mathrm{MSD}}$. Thus, the novel approach, based on the PCA, is expected to outperform the traditional approach, based on the MSD, in applications where few baseline data are available.

## 5. Conclusions

This paper presented an unsupervised learning vibration-based damage detection strategy for SHM applications where only few data are available to build the training set. In these cases, indeed, the whole variability of the damage feature due to operational and environmental conditions is not described in the training set. This leads to changes of the damage feature which can possibly either mask a damage or lead to false positives. The proposed SHM approach is based on the use of a damage index obtained through the PCA of the selected damage features. Indeed, relying on the assumption that under healthy reference conditions the variability of the collected damage features is only due to environmental and operational variations, these variations will affect the first few PCs, which explain most of the variability in the data. Thus, by discarding these few PCs, the remaining ones are not correlated to the environmental effects and can be used to define a temperature-insensitive damage index.

The effectiveness of the proposed approach was proved on both simulated and experimental data related to an axially loaded beam-like structure and considering the first bending natural frequencies as a multivariate damage feature. In both the cases, the proposed approach was compared with the MSD-based outlier detection method, widely adopted in unsupervised learning SHM literature. The simulations allowed highlighting the behaviour of the method when seasonal temperature trends are present. Both strategies showed similar performances when a complete temperature cycle is contained in the baseline set. Conversely, by reducing the baseline, and thus limiting the temperature conditions included in the training set, the PCA-based damage index outperformed the MSD-based one. It, indeed, did not produce any false positive and showed a higher sensitivity to damage, even when only a quarter of the simulated seasonal trend was included in the training set. Moreover, unlike the MSD-based approach, the PCA-based one successfully identified the smallest damage which was intentionally introduced in the experimental set-up. The experimental campaign proved the PCA-based method robustness, sensitivity and effectiveness in presence of real and uncontrolled temperature conditions.

It is worth mentioning that, when a damage is introduced in the structure, a transient of the PCA-based damage index is noticed. Although the effect of the damage can be clearly detected even during the transient, it may represent a limit of the approach. Thus, future studies will be devoted to the investigation of the effect of some parameters (e.g., the length of the new data added to the training set and used to calculate the damage index) on the transient duration and on the method sensitivity. Moreover, also the number of PCs to discard in the damage index evaluation is worthy of a deeper analysis. Future studies could, indeed, allow for an automated strategy able to define the PCs which have to be neglected in the damage index evaluation. The proposed approach, together with the future studies on its optimisation, will constitute a step forward in the monitoring of all those structures where long training is not possible and whose most relevant damage features are also the most sensitive to environmental and operating conditions.

## Figures and Tables

**Table 1:** Parameters of the simulated tie-rod.

*L* [m]	$S_{0}$ [N]	*E* [GPa]	ρ [kg/m^3^]	*w* [m]	*h* [m]	*k* [N/°C]
4	8 × 10^3^	69	2.7 × 10^3^	1.5 × 10^−2^	2.5 × 10^−2^	−60

**Table 2:** List of $\Delta f_{i}$ values used to simulate damage.

Young’s Modulus Reduction [%]	$\Delta f_{1}$ [%]	$\Delta f_{2}$ [%]	$\Delta f_{3}$ [%]	$\Delta f_{4}$ [%]	$\Delta f_{5}$ [%]
10	1.0 × 10^−2^	1.4 × 10^−4^	5.4 × 10^−2^	4.4 × 10^−4^	8.6 × 10^−2^
30	3.9 × 10^−2^	5.6 × 10^−4^	2.0 × 10^−1^	1.7 × 10^−3^	3.2 × 10^−1^

**Table 3:** Simulated test cases.

Test Case	Damage	*T* Cycle	*b*	Total Number of Samples
sim 1	No	Long	4320 $\left(b_{1}\right)$	8640
sim 2	No	Long	1008 $\left(b_{2}\right)$	8640
sim 3	No	Long + short	1008 $\left(b_{2}\right)$	8640
sim 4	Yes	Long + short	4320 $\left(b_{1}\right)$	21600
sim 5	No	Real	1008 $\left(b_{2}\right)$	12960
sim 6	No	Real	4320 $\left(b_{1}\right)$	12960
sim 7	Yes	Real	4320 $\left(b_{1}\right)$	12960
sim 8	Yes	Real	1008 $\left(b_{2}\right)$	12960

**Table 4:** Tie-rod eigenfrequencies of the first six bending vertical modes, identified after the tensioning procedure.

$f_{1}$ [Hz]	$f_{2}$ [Hz]	$f_{3}$ [Hz]	$f_{4}$ [Hz]	$f_{5}$ [Hz]	$f_{6}$ [Hz]
13.89	30.98	53.36	81.82	116.55	157.95

## Data Availability

The data presented in this study are available on request from the corresponding author.

## References

[1] Farrar C.R., Worden K. (2007). An introduction to structural health monitoring. Philos. Trans. R. Soc. A Math. Phys. Eng. Sci..

[2] Farrar C.R., Worden K. (2012). Structural Health Monitoring: A Machine Learning Perspective.

[3] Fan W., Qiao P. (2011). Vibration-based damage identification methods: A review and comparative study. Struct. Health Monit..

[4] Hou R., Xia Y. (2021). Review on the new development of vibration-based damage identification for civil engineering structures: 2010–2019. J. Sound Vib..

[5] Avci O., Abdeljaber O., Kiranyaz S., Hussein M., Gabbouj M., Inman D.J. (2021). A review of vibration-based damage detection in civil structures: From traditional methods to Machine Learning and Deep Learning applications. Mech. Syst. Signal Process..

[6] Brownjohn J.M., de Stefano A., Xu Y.L., Wenzel H., Aktan A.E. (2011). Vibration-based monitoring of civil infrastructure: Challenges and successes. J. Civ. Struct. Health Monit..

[7] Limongelli M.P., Manoach E., Quqa S., Giordano P.F., Bhowmik B., Pakrashi V., Cigada A. (2021). Vibration Response-Based Damage Detection. Springer Aerospace Technology.

[8] Entezami A., Shariatmadar H., Karamodin A. (2019). Data-driven damage diagnosis under environmental and operational variability by novel statistical pattern recognition methods. Struct. Health Monit..

[9] Entezami A., Sarmadi H., Behkamal B., Mariani S. (2020). Big data analytics and structural health monitoring: A statistical pattern recognition-based approach. Sensors.

[10] Razavi B.S., Mahmoudkelayeh M.R., Razavi S.S. (2021). Damage identification under ambient vibration and unpredictable signal nature. J. Civ. Struct. Health Monit..

[11] Tran T.T., Ozer E. (2020). Automated and model-free bridge damage indicators with simultaneous multiparameter modal anomaly detection. Sensors.

[12] Farrar C.R., Doebling S.W., Nix D.A. (2001). Vibration-based structural damage identification. Philos. Trans. R. Soc. A Math. Phys. Eng. Sci..

[13] Chen H.P., Ni Y.Q. (2018). Vibration-Based Damage Identification Methods. Struct. Health Monit. Large Civ. Eng. Struct..

[14] Sohn H. (2007). Effects of environmental and operational variability on structural health monitoring. Philos. Trans. R. Soc. A Math. Phys. Eng. Sci..

[15] Peeters B., Maeck J., De Roeck G. (2004). Vibration-based damage detection in civil engineering: Excitation sources and temperature effects. Noise Vib. Worldw..

[16] Alampalli S. (2000). Effects of testing, analysis, damage, and environment on modal parameters. Mech. Syst. Signal Process..

[17] Collini L., Garziera R., Riabova K. (2020). Detection of cracks in axially loaded tie-rods by vibration analysis. Nondestruct. Test. Eval..

[18] Lucà F., Manzoni S., Cigada A., Frate L. (2022). A vibration-based approach for health monitoring of tie-rods under uncertain environmental conditions. Mech. Syst. Signal Process..

[19] Pereira S., Magalhães F., Gomes J.P., Cunha Á., Lemos J.V. (2021). Vibration-based damage detection of a concrete arch dam. Eng. Struct..

[20] Hu W.H., Tang D.H., Teng J., Said S., Rohrmann R.G. (2018). Structural health monitoring of a prestressed concrete bridge based on statistical pattern recognition of continuous dynamic measurements over 14 years. Sensors.

[21] Peeters B., Roeck G.D. (2015). One-year monitoring of the Z24Bridge: Environmental effects versus damage events. Earthq. Eng. Struct. Dyn..

[22] Cross E.J., Koo K.Y., Brownjohn J.M., Worden K. (2013). Long-term monitoring and data analysis of the Tamar Bridge. Mech. Syst. Signal Process..

[23] Torzoni M., Rosafalco L., Manzoni A., Mariani S., Corigliano A. (2022). SHM under varying environmental conditions: An approach based on model order reduction and deep learning. Comput. Struct..

[24] Zhou H.F., Ni Y.Q., Ko J.M. (2010). Constructing input to neural networks for modeling temperature-caused modal variability: Mean temperatures, effective temperatures, and principal components of temperatures. Eng. Struct..

[25] Shan W., Wang X., Jiao Y. (2018). Modeling of Temperature Effect on Modal Frequency of Concrete Beam Based on Field Monitoring Data. Shock Vib..

[26] Ni Y.Q., Hua X.G., Fan K.Q., Ko J.M. (2005). Correlating modal properties with temperature using long-term monitoring data and support vector machine technique. Eng. Struct..

[27] Kromanis R., Kripakaran P. (2013). Support vector regression for anomaly detection from measurement histories. Adv. Eng. Inform..

[28] Kullaa J. (2011). Distinguishing between sensor fault, structural damage, and environmental or operational effects in structural health monitoring. Mech. Syst. Signal Process..

[29] Erazo K., Sen D., Nagarajaiah S., Sun L. (2019). Vibration-based structural health monitoring under changing environmental conditions using Kalman filtering. Mech. Syst. Signal Process..

[30] Kullaa J. (2022). Damage detection and localization under variable environmental conditions using compressed and reconstructed bayesian virtual sensor data. Sensors.

[31] Kullaa J. (2020). Robust damage detection in the time domain using Bayesian virtual sensing with noise reduction and environmental effect elimination capabilities. J. Sound Vib..

[32] Maes K., Van Meerbeeck L., Reynders E.P., Lombaert G. (2022). Validation of vibration-based structural health monitoring on retrofitted railway bridge KW51. Mech. Syst. Signal Process..

[33] Sen D., Erazo K., Zhang W., Nagarajaiah S., Sun L. (2019). On the effectiveness of principal component analysis for decoupling structural damage and environmental effects in bridge structures. J. Sound Vib..

[34] Soo Lon Wah W., Chen Y.T., Roberts G.W., Elamin A. (2018). Separating damage from environmental effects affecting civil structures for near real-time damage detection. Struct. Health Monit..

[35] Deraemaeker A., Worden K. (2018). A comparison of linear approaches to filter out environmental effects in structural health monitoring. Mech. Syst. Signal Process..

[36] Cross E.J., Manson G., Worden K., Pierce S.G. (2012). Features for damage detection with insensitivity to environmental and operational variations. Proc. R. Soc. A Math. Phys. Eng. Sci..

[37] Surace C., Bovsunovsky A. (2020). The use of frequency ratios to diagnose structural damage in varying environmental conditions. Mech. Syst. Signal Process..

[38] Martakis P., Reuland Y., Imesch M., Chatzi E. (2022). Reducing uncertainty in seismic assessment of multiple masonry buildings based on monitored demolitions. Bull. Earthq. Eng..

[39] Campagnari S., Di Matteo F., Manzoni S., Scaccabarozzi M., Vanali M. (2017). Estimation of axial load in tie-rods using experimental and operational modal analysis. J. Vib. Acoust. Trans. ASME.

[40] Kernicky T., Whelan M., Al-Shaer E. (2018). Dynamic identification of axial force and boundary restraints in tie rods and cables with uncertainty quantification using Set Inversion Via Interval Analysis. J. Sound Vib..

[41] Rainieri C., Fabbrocino G. (2015). Development and validation of an automated operational modal analysis algorithm for vibration-based monitoring and tensile load estimation. Mech. Syst. Signal Process..

[42] Resta C., Chellini G., Falco A.D. (2020). Dynamic assessment of axial load in tie-rods by means of acoustic measurements. Buildings.

[43] De Falco A., Resta C., Sevieri G. (2021). Sensitivity analysis of frequency-based tie-rod axial load evaluation methods. Eng. Struct..

[44] Coïsson E., Collini L., Ferrari L., Garziera R., Riabova K. (2019). Dynamical Assessment of the Work Conditions of Reinforcement Tie-Rods in Historical Masonry Structures. Int. J. Archit. Herit..

[45] Cescatti E., Da Porto F., Modena C. (2019). Axial Force Estimation in Historical Metal Tie-Rods: Methods, Influencing Parameters, and Laboratory Tests. Int. J. Archit. Herit..

[46] Garziera R., Amabili M., Collini L. (2011). A hybrid method for the nondestructive evaluation of the axial load in structural tie-rods. Nondestruct. Test. Eval..

[47] Tullini N., Rebecchi G., Laudiero F. (2019). Reliability of the tensile force identification in ancient tie-rods using one flexural mode shape. Int. J. Archit. Herit..

[48] Makoond N., Pelà L., Molins C. (2022). Robust estimation of axial loads sustained by tie-rods in historical structures using Artificial Neural Networks. Struct. Health Monit..

[49] Lucà F., Manzoni S., Cerutti F., Cigada A. (2022). A Damage Detection Approach for Axially Loaded Beam-like Structures Based on Gaussian Mixture Model. Sensors.

[50] Lucà F., Manzoni S., Cigada A., Barella S., Gruttadauria A., Cerutti F. (2022). Automatic Detection of Real Damage in Operating Tie-Rods. Sensors.

[51] Gentile C., Poggi C., Ruccolo A., Vasic M. (2019). Vibration-Based Assessment of the Tensile Force in the Tie-Rods of the Milan Cathedral. Int. J. Archit. Herit..

[52] Jolliffe I.T. (2002). Principal Component Analysis.

[53] Datteo A., Lucà F., Busca G., Cigada A. (2017). Long-time monitoring of the G. Meazza stadium in a pattern recognition prospective. Procedia Eng..

[54] Lucà F., Manzoni S., Cigada A. (2023). Data Driven Damage Detection Strategy Under Uncontrolled Environment. European Workshop on Structural Health Monitoring.

[55] Worden K., Manson G., Fieller N.R. (2000). Damage detection using outlier analysis. J. Sound Vib..

[56] Gallego G., Cuevas C., Mohedano R., García N. (2013). On the mahalanobis distance classification criterion for multidimensional normal distributions. IEEE Trans. Signal Process..

[57] Cheli F., Diana G. (2015). Advanced Dynamics of Mechanical Systems.

[58] Valle J., Fernández D., Madrenas J. (2019). Closed-form equation for natural frequencies of beams under full range of axial loads modeled with a spring-mass system. Int. J. Mech. Sci..

[59] Galef A.E. (1968). Bending Frequencies of Compressed Beams. J. Acoust. Soc. Am..

[60] Ewins D.J. (2001). Modal Testing: Theory, Practice and Application.

[61] Peeters B., Van Der Auweraer H., Guillaume P., Leuridan J. (2004). The PolyMAX frequency-domain method: A new standard for modal parameter estimation?. Shock Vib..

[62] Sakaris C.S., Sakellariou J.S., Fassois S.D. (2017). Random-vibration-based damage detection and precise localization on a lab–scale aircraft stabilizer structure via the Generalized Functional Model Based Method. Struct. Health Monit..

[63] Sarrafi A., Mao Z., Niezrecki C., Poozesh P. (2018). Vibration-based damage detection in wind turbine blades using Phase-based Motion Estimation and motion magnification. J. Sound Vib..

[64] Banerjee S., Ricci F., Monaco E., Mal A. (2009). A wave propagation and vibration-based approach for damage identification in structural components. J. Sound Vib..

[65] Yi W.J., Zhou Y., Kunnath S., Xu B. (2008). Identification of localized frame parameters using higher natural modes. Eng. Struct..

